# Is Coronary Artery Ectasia a Progressive Disease? A Self-Controlled Retrospective Cohort Study

**DOI:** 10.3389/fcvm.2021.774597

**Published:** 2021-12-06

**Authors:** Ruifeng Liu, Huiqiang Zhao, Xiangyu Gao, Siwen Liang

**Affiliations:** Department of Cardiology, Beijing Friendship Hospital Affiliated to Capital Medical University, Beijing, China

**Keywords:** coronary artery ectasia (CAE), coronary angiogram (CAG), progression, Gensini score, atherosclerotic change

## Abstract

**Objective:** It is essential to understand whether coronary artery ectasia (CAE) progresses over time because the patients might be under the risk of coronary rupture, and stent implant should be avoided if ectatic changes progress.

**Methods:** A consecutive series of 99 CAE patients who had undergone coronary angiography at least twice were enrolled and followed up for 1–16 years until they received a second angiogram. Subjects were divided into two groups (1–5 vs. 5–16 years of follow-up), then the basic clinical characteristics and coronary artery images were compared over time.

**Results:** (1) All CAE patients exhibited atherosclerosis, and a majority presented with acute myocardial infarction. Most baseline clinical characteristics were relatively stable. (2) Atherosclerosis (indicated by the distribution of stenosis in coronary vessels) and the Gensini scores progressed significantly. Ectasia extent showed minimal changes as indicated by blood vessel involvement, Markis type, coronary blood flow, ectasia diameter, and ectasia length. (3) Multilinear regression analysis revealed that the underlying factors related to stenosis evolution indicated by fold of Gensini score were: longer time interval, lower baseline Gensini score, and higher hypersensitive C-reactive protein concentration. (4) There was a relationship between the ectatic diameter and the extent of stenosis.

**Conclusions:** For CAE patients with atherosclerosis followed for 1–16 years, there was minimal CAE progression, while the atherosclerosis progressed and the ectasia extent was related to degree of stenosis. The results indicate that prevention and treatment of atherosclerotic changes might have more clinical significance than addressing ectatic changes.

## Introduction

Coronary artery ectasia (CAE) has been defined as an ectatic artery diameter more than 1.5 times greater than the diameters of the adjacent normal segments (1). Its prevalence is 0.3–4.9% in patients who have undergone coronary angiography (CAG) (1). Its incidence increases with age, and the average diagnosis occurs at 55 ± 10 years old (2). The current understanding of CAE is still relatively limited. More than 80% of CAE patients experience coronary artery disease (CAD) (1, 3); their main symptoms include angina, myocardial infarction, arrhythmia, and sudden death, and most are treated under the principle of current guidelines for CAD (4, 5). However, CAE is also characterized by obvious slow blood flow in dilated coronary arteries (6, 7), increased inflammatory markers in peripheral blood (8), anomalies in other blood vessels (9–11), and decreased left ventricular diastolic function (12). Pathologically, CAE is characterized by the extensive destruction of the musculoelastic elements in the coronary wall (3, 4). Possible etiologies include enzyme destruction (2), vascular endothelial dysfunction and oxidative stress (13), and genetic factors (14). It was unclear whether CAE was progressive disease until recently, and in clinical practice one of the major concerns is that CAE patients might be at risk of continuous rapid expansion even the coronary rupture like giant coronary aneurysm (another major concern was thrombotic event) (15), and stent implant in the coronary artery should be avoided if ectatic changes progress. This self-controlled retrospective cohort study evaluated patient medical records and images from two separate CAG sessions to evaluate dynamic changes of CAE over time.

## Methods

### Subjects

A consecutive series of 99 subjects were enrolled from 129 CAE patients who had undergone at least two CAGs at the Cardiac Catheterization Center of Beijing Friendship Hospital between 2001 and 2021. There was no mortality between the twice CAGs. Twenty-one patients did not meet the inclusion and exclusion criteria, and seven were excluded because they had undergone coronary artery bypass grafting. The subjects were divided into two groups based on the follow-up time: 1–5 years of follow-up (≥1 and <5 years, overall time interval time was 3.04 [2.48–4.17] years) and 5–16 years of follow-up (≥5 years, overall interval time was 8.15 [6.52–11.48] years). The mean ages for Groups 1 and 2 were 61.62 ± 10.69 (42–79 years old) and 59.52 ± 11.24 (36–73 years old), respectively. The two groups were analyzed separately. Age, sex, basic clinical characteristics, patients' inflammatory condition, echocardiographic measurements, and coronary artery imaging features were compared at the receipt of the first and second CAGs.

### Cohort Description

This study was approved by the ethics committee of Beijing Friendship Hospital Affiliated to Capital Medical University and was in accordance with the Declaration of Helsinki (P2-013-02). Informed consent was not required because it was a retrospective study with no additional medical interventions. Most of the costs of disease evaluation and medical interventions were covered by the medical insurance provided by Chinese government.

### Inclusion and Exclusion Criteria

Each angiogram was interpreted by two independent cardiologists. CAE was defined as an ectatic artery diameter more than 1.5 times the diameters of the adjacent normal segments. The exclusion criteria were cardiomyopathy, valvular heart disease, heart failure, collagen tissue disease, vasculitis, syphilis, chronic obstructive lung disease, pulmonary hypertension, early menopause, documented history of hepatic disease, renal failure, known malignancy, local or systemic infection, other inflammatory diseases, and previous history of infection (<3 months). To ensure data reliability, we excluded 7 patients who had been treated with coronary artery bypass grafting at any time because the original coronary vessel would degenerate rapidly after this operation according to our clinical experiences. There was no Kawasaki disease in this cohort.

### Medical Records

Most of the study data were extracted from hospital medical records that were detailed and intact. The obtained data included demographics (age and sex), disease history (e.g., CAD and diabetes), presence of smoking or drinking, family history (hypertension, diabetes, and CAD), body mass index (BMI), and pre-admission medications. Most laboratory detection markers were measured on the second day after admission. Left ventricular ejection fraction was evaluated by transthoracic echocardiography.

### Coronary Artery Imaging Features

CAG was performed *via* a Radical approach or Femoral artery approach without the use of adenosine or a calcium channel blocker. In the Markis classification system (4), type I refers to diffuse ectasia in two or three vessels, type II indicates diffuse ectasia in one vessel and localized ectasia in another, type III refers to diffuse ectasia in only one vessel, and type IV indicates localized or segmental ectasia. Thrombolysis in myocardial infarction frame counts in the three major coronary arteries (16) and Gensini scores (17) were visually determined by experienced interventional cardiologists on the basis of specified anatomic landmarks and imaging features. To facilitate comparisons between the first and second CAGs, the first Gensini score was evaluated after revascularization (thrombolysis, percutaneous coronary intervention) if performed.

### Statistical Analysis

This self-controlled retrospective cohort study was conducted through comparisons of the medical data from the first and second CAGs in a consecutive series of CAE patients. IBM SPSS Statistics, Version 25.0 (IBM Corp., Armonk, NY, USA) was used in the data analysis. The data were initially analyzed with the Kolmogorov–Smirnov test to assess normality. Normally distributed continuous data are presented as mean ± standard deviation. Non-Gaussian distributions are presented as median and interquartile range. Paired *t*-tests and Wilcoxon signed-rank tests were used for the bivariate analyses of normally and non-normally distributed continuous data, respectively. For non-parametric data, the percentages were compared by chi-square or Fisher's exact test when appropriate. Multiple linear regression for changes in Gensini scores were performed by the backward stepwise method and a threshold of 0.05. Pearson correlation coefficients were calculated to determine the ectasia and Gensini score changes. Differences were considered significant at *p* < 0.05.

## Results

Table 1 presents the basic CAE patient characteristics at the time of the first and second CAGs. All of the CAE patients in both groups exhibited atherosclerosis, and most presented with acute myocardial infarction (AMI). The blood pressure and lipid concentration measurements indicated decreasing tendencies in both groups. However, statistical significance was observed in only the 5–16-year follow-up group. The treatment information for all study subjects is provided. A subset of patients underwent revascularization with stent implantation (Supplementary Material).

**Table 1:** Baseline characteristics of coronary artery ectasia patients.

	**Interval 1–5 years (*****n*** **=** **53)**			**Interval 5–16 years (*****n*** **=** **46)**		
	**Baseline CAG**	**Second CAG**	***p-*value**	**Baseline CAG**	**Second CAG**	***p-*value**
Sex, male (%)	41 (77.36%)	41 (77.36%)	–	35 (76.09%)	35 (76.09%)	–
Age (years)	61.62 ± 10.69	64.33 ± 10.41	0.000	59.52 ± 11.24	68.65 ± 11.68	0.000
Stable CHD, *n* (%)	6 (11.32%)	4 (7.55%)	0.468	10 (21.74%)	5 (10.87%)	0.055
Unstable angina pectoris, *n* (%)	29 (54.72%)	36 (67.92%)		16 (34.78%)	29 (63.04%)	
NSTEMI, *n* (%)	10 (18.87%)	9 (16.98%)		10 (21.74%)	7 (15.22%)	
STEMI, *n* (%)	8 (15.09%)	4 (7.55%)		10 (21.74%)	5 (10.87%)	
OMI history, *n* (%)	10 (18.87%)	15 (28.30%)	0.180	9 (19.57%)	12 (26.09%)	0.320
Hypertension, *n* (%)	40 (75.47%)	40 (75.47%)	–	28 (60.87%)	30 (65.22%)	–
SBP (mmHg)	128.48 ± 19.27	124.86 ± 14.90	0.228	135.95 ± 18.33	127.88 ± 16.63	0.026
DBP (mmHg)	78.26 ± 12.56	74.52 ± 10.32	0.081	79.67 ± 12.04	73.48 ± 9.10	0.005
Heart rate (per minute)	66.00 (72.00–78.00)	60.75 (68.00–75.00)	0.222	63.75 (70.00–78.50)	64.00 (70.00–74.25)	0.334
BMI (kg/m^2^)	26.71 ± 2.80	26.67 ± 2.74	0.925	25.77 ± 2.37	26.72 ± 3.17	0.176
Diabetes, *n* (%)	15 (28.30%)	13 (24.53%)	0.413	13 (28.26%)	15 (32.61%)	0.650
Smoking, *n* (%)	28 (52.83%)	32 (60.38%)	0.278	26 (56.52%)	27 (58.70%)	0.883
Alcohol, *n* (%)	24 (45.28%)	21 (39.62%)	0.347	21 (45.65%)	21 (45.65%)	1.000
CAD family history, *n* (%)	16 (30.19%)	21 (39.62%)	0.208	4 (8.70%)	17 (37.83%)	0.001
Hypertension family history, *n* (%)	18 (33.96%)	23 (43.40%)	0.425	4 (8.70%)	9 (19.57%)	0.135
Diabetes family history, *n* (%)	3 (5.66%)	5 (9.43%)	0.479	2 (4.35%)	4 (8.70%)	0.398
ALT (U/L)	13.00 (19.00–30.00)	10.00 (17.00–29.00)	0.655	15.00 (19.00–36.00)	13.00 (18.10–28.00)	0.072
AST (U/L)	17.00 (25.00–50.00)	16.00 (22.00–29.40)	0.004	16.50 (24.00–37.50)	15.75 (19.00–25.58)	0.001
Urea nitrogen	4.40 (5.29–6.54)	4.96 (5.64–7.09)	0.530	4.69 (6.12–7.92)	4.51 (5.42–6.58)	0.016
Creatinine	66.20 (83.00–96.50)	68.25 (82.60–89.60)	0.073	68.08 (84.18–99.78)	65.90 (82.50–93.20)	0.582
TC, mmol/L	4.47 ± 1.46	3.80 ± 0.84	0.002	4.67 ± 1.27	3.84 ± 1.21	0.001
TG, mmol/L	1.97 ± 1.51	1.62 ± 1.08	0.115	1.83 ± 1.54	1.88 ± 1.27	0.857
HDL-c, mmol/L	1.00 ± 0.24	1.06 ± 0.23	0.044	0.92 ± 0.18	0.99 ± 0.22	0.061
LDL-c, mmol/L	2.58 ± 0.89	2.01 ± 0.59	0.000	2.44 ± 0.66	2.16 ± 0.90	0.237
Blood sugar, mmol/L	5.01 (5.44–6.60)	4.75 (5.56–7.41)	0.961	5.12 (5.90–7.84)	4.87 (5.70–7.37)	0.950
HbA1c, %	6.30 ± 1.36	6.55 ± 1.05	0.388	6.47 ± 0.74	7.60 ± 1.21	0.404
Antiplatelet, *n* (%)	52 (98.11%)			44 (95.65%)		
Statins, *n* (%)	53 (100.00%)			45 (97.83%)		
ACEI/ARB, *n* (%)	40 (75.47%)			38 (82.61%)		
β-blocker, *n* (%)	48 (90.57%)			43 (93.48%)		
CCB, *n* (%)	28 (52.83%)			23 (50.00%)		
Nitrate, *n* (%)	32 (60.38%)			36 (78.26%)		
Revascularization^*^	33 (62.26%)			29 (63.04%)		

*The p-values for the comparisons of the results of the first and second coronary angiograms. CHD, coronary heart disease; NSTEMI, non-ST-segment elevation myocardial infarction; STEMI, ST-segment elevation myocardial infarction; OMI, old myocardial infarction; SBP, systolic blood pressure; DBP, diastolic blood pressure; BMI, body mass index; HbA1c, hemoglobin A1c; TC, total cholesterol; TG, triglyceride; HDL-c, high-density lipoprotein cholesterol; LDL-c, low-density lipoprotein cholesterol; ALT, alanine aminotransferase; BUN, blood urea nitrogen; AST, glutamic-oxaloacetic transaminase; ACEI/ARB, angiotensin-converting enzyme inhibitors and angiotensin receptor blockers; CCB, calcium channel antagonists*.

* *There were 62 CAE patients received stent implant, 54 of them received 1 stent, 5 of them received 2 stents, 2 of them received 3 stents, 1 of them received 4 stents. The average diameter (mm) and length (mm) of stents were: (1) left anterior descending branch (LAD), proximal segment, 3.27, 22.04; middle segment, 2.99, 26.47; distal segment, 3.00, 18.00; (2) left circumflex branch (LCX), proximal segment, 3.14, 25.71; distal segment, 2.68, 22.71; obtuse marginal branch (OM), 2.68, 16.80; right coronary artery (RCA), proximal segment 4.00, 28.00; middle segment, 3.10, 26.20; distal segment, 2.69, 25.60; posterior descending branches (PD), 2.67, 18.00; posterior branches of left ventricular (PL), 3.13, 19.50*.

The data in Table 2 show no obvious changes in the inflammation indicators for either group. CAG evaluations revealed increases in the left atrium dimensions and fractional shortening percentages, but no other characteristics were different over time.

**Table 2:** Inflammatory indicators and echocardiographic measurements.

	**Interval 1–5 years (*****n*** **=** **53)**			**Interval 5–16 years (*****n*** **=** **46)**		
	**Baseline CAG**	**Second CAG**	***p-*value**	**Baseline CAG**	**Second CAG**	***p-*value**
Leukocytes (10^3^/μL)	7.00 ± 1.88	6.75 ± 2.22	0.256	7.42 ± 2.54	6.92 ± 1.56	0.201
Neutrophils (10^3^/μL)	4.27 ± 1.29	4.44 ± 1.82	0.364	4.62 ± 2.46	4.65 ± 1.17	0.951
Lymphocyte (10^3^/μL)	1.98 ± 0.72	1.79 ± 0.75	0.040	1.83 ± 0.77	1.70 ± 0.60	0.335
NL ratio	2.30 ± 0.75	2.43 ± 1.07	0.555	2.79 ± 1.73	2.12 ± 0.51	0.024
Platelet (10^3^/μL)	225.80 ± 57.21	210.96 ± 55.80	0.015	198.58 ± 46.31	185.48 ± 37.72	0.090
MPV, fL	9.15 ± 1.22	9.58 ± 1.97	0.166	9.38 ± 1.75	9.20 ± 1.19	0.523
ESR, mm/h	5.00 (8.00–14.00)	6.00 (8.00–17.00)	0.414	0.00 (5.00–11.50)	3.50 (8.00–20.00)	0.180
hs-CRP, mg/L	0.95 (3.33–7.06)	0.66 (1.37–3.32)	0.047	1.01 (2.63–6.98)	0.80 (2.07–5.16)	0.084
E/A ratio	0.94 ± 0.33	0.84 ± 0.33	0.103	0.79 ± 0.23	0.86 ± 0.31	0.219
LVEF, %	62.93 ± 10.37	62.24 ± 10.25	0.669	60.03 ± 11.61	58.00 ± 11.73	0.265
EDD, cm	5.27 ± 0.55	5.20 ± 0.63	0.396	5.39 ± 0.77	5.40 ± 0.62	0.855
ESD, cm	3.47 ± 0.68	3.43 ± 0.69	0.610	3.69 ± 0.83	3.69 ± 0.77	0.988
EDV, ml	132.43 ± 35.77	134.80 ± 36.02	0.572	139.13 ± 37.23	143.50 ± 34.35	0.399
ESV, ml	54.64 ± 25.06	52.57 ± 27.01	0.453	58.19 ± 27.12	59.47 ± 29.63	0.787
SV, ml	82.48 ± 15.46	83.03 ± 15.47	0.828	85.10 ± 19.15	84.74 ± 15.26	0.898
LA, cm	3.67 ± 0.40	3.87 ± 0.46	0.009	3.55 ± 0.58	3.94 ± 0.43	0.000
AV, cm	1.85 ± 0.22	1.96 ± 0.49	0.177	1.94 ± 0.40	1.85 ± 0.27	0.241
RV, cm	1.76 ± 0.26	1.68 ± 0.34	0.151	1.62 ± 0.31	1.69 ± 0.34	0.300
LVPW amplitude, cm	0.97 ± 0.24	0.95 ± 0.21	0.629	0.91 ± 0.21	0.93 ± 0.22	0.723
LVPW thickness, cm	0.99 ± 0.12	0.97 ± 0.11	0.948	0.96 ± 0.12	0.97 ± 0.09	0.706
IVS amplitude, cm	0.84 ± 0.22	0.89 ± 0.20	0.249	0.79 ± 0.23	0.82 ± 0.27	0.511
IVS thickness	1.07 ± 0.18	1.05 ± 0.19	0.440	1.04 ± 0.16	1.05 ± 0.13	0.788
FS, %	9.28 ± 15.51	1.67 ± 6.28	0.001	14.94 ± 17.39	0.33 ± 0.08	0.000
Aorta amplitude, cm	0.68 ± 0.23	0.71 ± 0.23	0.590	0.68 ± 0.19	0.69 ± 0.22	0.804
Aorta inner diameter, cm	3.52 ± 0.51	3.54 ± 0.44	0.757	3.61 ± 0.42	3.58 ± 0.30	0.568
Ascending aorta, cm	3.73 ± 0.38	3.64 ± 0.45	0.083	3.67 ± 0.38	3.68 ± 0.38	0.803

*The p-values for the comparisons of the first and second coronary angiograms. AV, aortic valve diameter; E/A, E peak value to A peak value; EDD, end-diastolic dimension; EDV, end-diastolic volume; ESD, end-systolic dimension; ESR, erythrocyte sedimentation rate; ESV, end-systolic volume; FS, fractional shortening; hs-CRP, hypersensitive C-reactive protein; IVS, interventricular septum; LA, left atrium dimension; LVEF, left ventricular ejection fraction; LVPW, left ventricular posterior wall; MPV, mean platelet volume; NLR, neutrophil-to-lymphocyte ratio; RV, right ventricle dimension; SV, stroke volume*.

Table 3 shows the coronary imaging characteristics of the enrolled subjects. The lack of change in the extent of ectasia was indicated by blood vessel involvement, Markis type, ectatic diameter, ectatic length, and corrected thrombolysis in the myocardial infarction frame count. The Gensini scores and blood vessel involvement assessment revealed increases in the extent of stenosis over time.

**Table 3:** Coronary evaluations for ectasia and stenosis.

	**Interval 1–5 years (*****n*** **=** **53)**			**Interval 5–16 years (*****n*** **=** **46)**		
	**Baseline CAG**	**Second CAG**	***p*-value**	**Baseline CAG**	**Second CAG**	***p*-value**
LM ectasia, *n* (%)	5 (9.43%)	5 (9.43%)	0.629	3 (6.52%)	3 (6.52%)	0.662
LAD ectasia, *n* (%)	13 (24.53%)	15 (28.30%)	0.413	8 (17.39%)	10 (21.74%)	0.397
LCX ectasia, *n* (%)	18 (33.96%)	19 (35.85%)	0.500	19 (41.30%)	20 (43.48%)	0.415
RCA ectasia, *n* (%)	38 (71.70%)	39 (73.58%)	0.500	29 (63.04%)	31 (67.39%)	0.862
Markis I, *n* (%)	5 (9.43%)	4 (7.55%)	0.577	4 (8.70%)	5 (10.87%)	0.923
Markis II, *n* (%)	5 (9.43%)	10 (18.87%)		4 (8.70%)	3 (6.52%)	
Markis III, *n* (%)	29 (54.72%)	26 (49.06%)		23 (50.00%)	25 (54.35%)	
Markis IV, *n* (%)	14 (26.42%)	13 (24.53%)		15 (32.61%)	13 (28.26%)	
Ectasia diameter, mm	4.50 (5.00–5.45)	4.45 (5.00–5.55)	0.713	4.50 (5.05–5.63)	4.60 (5.00–5.73)	0.424
Ectasia length, mm	8.00 (11.00–16.00)	8.30 (11.00–16.90)	0.711	7.00 (10.50–16.50)	7.68 (10.00–17.50)	0.845
Ectasia fold	1.52 (1.58–1.73)	1.51 (1.60–1.70)	0.996	1.53 (1.63–1.80)	1.56 (1.64–1.88)	0.381
CTFC	28.00 (32.00–36.00)	29.85 (32.00–36.50)	0.548	24.00 (30.00–40.00)	24.00 (32.00–40.00)	0.150
LM stenosis, *n* (%)	2 (3.77%)	2 (3.77%)	0.691	0 (0.00%)	1 (2.17%)	1.000
LAD stenosis, *n* (%)	43 (81.13%)	47 (88.68%)	0.208	28 (60.87%)	39 (84.78%)	0.010
LCX stenosis, *n* (%)	34 (64.15%)	42 (79.25%)	0.065	23 (50.00%)	38 (82.61%)	0.000
RCA stenosis, *n* (%)	36 (67.92%)	45 (84.91%)	0.033	20 (43.48%)	40 (86.96%)	0.000
Gensini score	15.00 (19.00–34.00)	18.50 (29.00–54.00)	0.000	16.00 (25.50–40.00)	28.75 (45.00–72.00)	0.000

*The p-values for the comparisons of the first and second coronary angiograms. CTFC, corrected thrombolysis in the myocardial infarction frame count; LAD, left anterior descending coronary artery; LM, left main coronary artery; LCX, left circumflex coronary artery; RCA, right coronary artery. The first Gensini score was evaluated after revascularization (thrombolysis, percutaneous coronary intervention) if received*.

**Figure 1:**
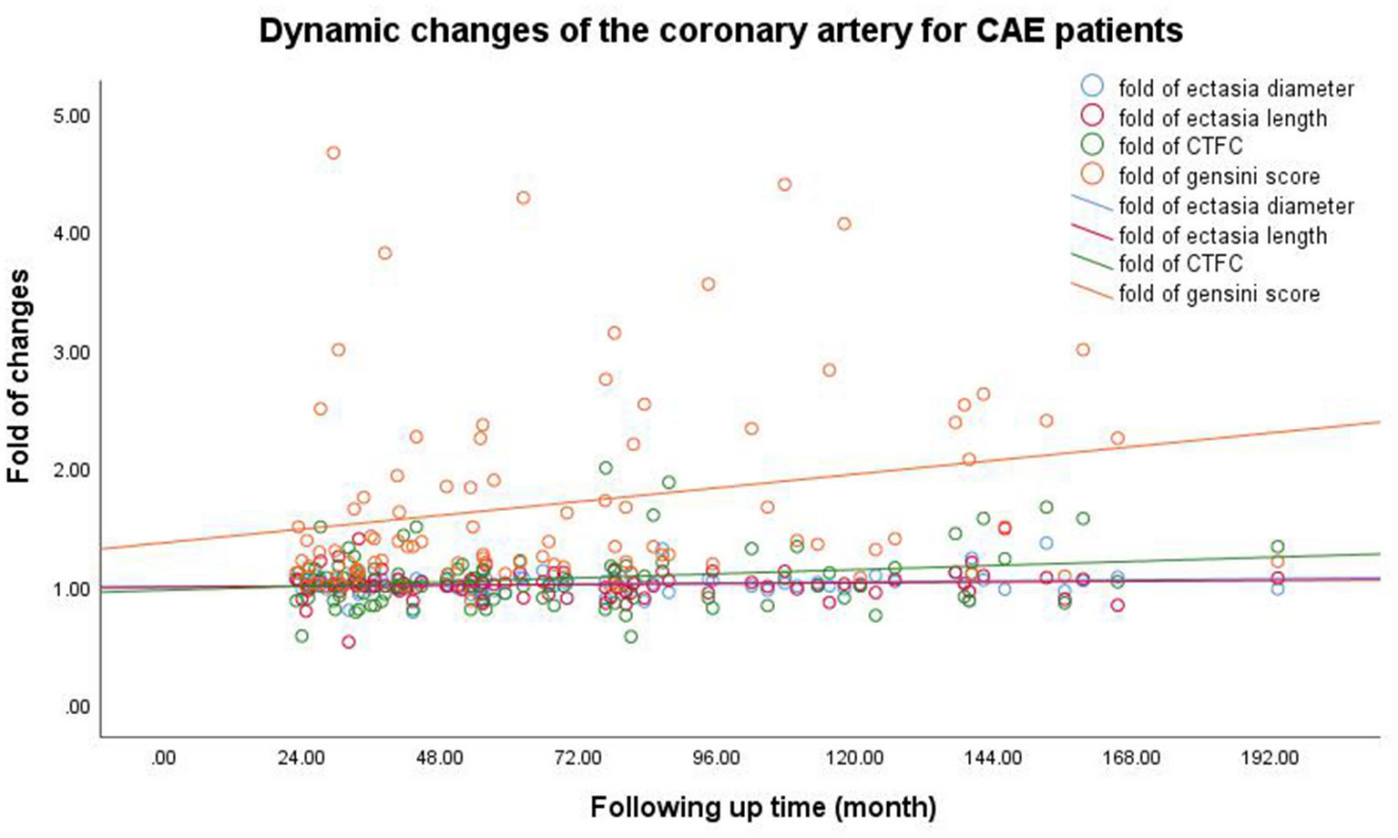
Dynamic changes of coronary artery for CAE patients. The Pearson correlation coefficients (*p*-values) for the relationship between follow-up time and the ectasia diameter, length, and extent and the Gensini scores were 0.138 (0.172), 0.053 (0.601), 0.201 (0.046), and 0.237 (0.021), respectively. CAE, coronary artery ectasia; CTFC, corrected thrombolysis in myocardial infarction frame count.

Figure 1 shows minimal changes in ectatic extent as indicated by the ectatic diameter and length. Coronary blood flow indicated by corrected thrombolysis in the myocardial infarction frame count showed an increasing tendency, and atherosclerotic progression was observed in all CAE patients. The detailed measurements of the first and second CAGs for all patients are listed in Table 3.

Table 4 shows the multiple linear regression results regarding the fold change of Gensini scores between the first and second CAGs (fold changes, equal to ratio of the second Gensini score to the first Gensini score). The results suggest that longer follow-up times, lower baseline Gensini score, and higher hypersensitive C-reactive protein concentration contributed to the increased Gensini scores.

**Table 4:** Results of Multiple linear regression for Gensini score fold changes.

**Items**	**Beta**	**Standardized beta coefficient**	**95% CI for beta**		***t*-value**	***p*-value**
Constant	1.257		0.850	1.664	6.171	0.000
Follow-up time (month)	0.005	0.228	0.000	0.010	2.023	0.047
Gensini score	−0.006	−0.217	−0.011	0.000	−1.936	0.057
hs-CRP, mg/L	0.046	0.286	0.010	0.083	2.539	0.014

*hs-CRP, hypersensitive C-reactive protein. In this multiple linear regression model, the dependent factor was Gensini score changes (fold change, equal to ratio of the second Gensini score to the first Gensini score) in the first and second CAGs. The independent factors selected from the first CAG were sex, age, hypertension, diabetes, smoking, alcohol, low-density lipoprotein cholesterol, hs-CRP, Markis classification, ectasia extent, baseline Gensini score, follow-up time, antiplatelet use, statins, angiotensin-converting enzyme inhibitors and angiotensin receptor blockers, beta blockers, calcium channel blockers, and nitrates*.

Table 5 shows the relationship between ectasia and stenosis. The right coronary artery (RCA) was most frequently involved in cases with a combination of ectasia and stenosis. On the second CAG, there were increases in the percentage of patients with both stenosis and ectasia in the RCA (all 99 affected) and in the ratio of vessels with both ectasia and stenosis to all abnormal vessels (dilation and/or atherosclerosis).

**Table 5:** Relationship between ectasia and stenosis.

	**LM**			**LAD**			**LCX**			**RCA**		
	**Baseline**	**Second**	***p*-value**	**Baseline**	**Second**	***p*-value**	**Baseline**	**Second**	***p*-value**	**Baseline**	**Second**	***p*-value**
Stenosis	2 (2.02%)	3 (3.03%)	0.651	74 (74.75%)	86 (86.87%)	0.009	60 (60.61%)	80 (80.81%)	0.000	56 (56.57%)	85 (85.86%)	0.000
Ectasia	8 (8.08%)	8 (8.08%)	1.000	21 (21.21%)	25 (25.25%)	0.501	36 (36.36%)	39 (39.39%)	0.660	67 (67.68%)	70 (70.71%)	0.664
Both stenosis and ectasia	0 (0.00%)	1 (1.01%)	0.316	18 (18.18%)	21 (21.21%)	0.470	26 (26.26%)	33 (33.33%)	0.158	35 (35.35%)	63 (63.64%)	0.000
Overlap rate	0.00%	18.18%	0.486	37.89%	37.84%	0.996	58.33%	55.46%	0.167	56.92%	81.30%	0.038

*The p-values for the comparisons of the first and second coronary angiograms. The significance level was 0.05. LAD, left anterior descending coronary artery; LCX, left circumflex coronary artery; LM, left main coronary artery; RCA, right coronary artery. “Both stenosis and ectasia” means vessels from CAE patients simultaneously presented with both dilation and atherosclerosis, the percentage number equal to ratio of this number to all 99 patients; Overlap rate means the ratio of numbers of vessels that simultaneously presented with both dilation and atherosclerosis to numbers of abnormal vessels with dilation and/or atherosclerosis*.

**Figure 2:**
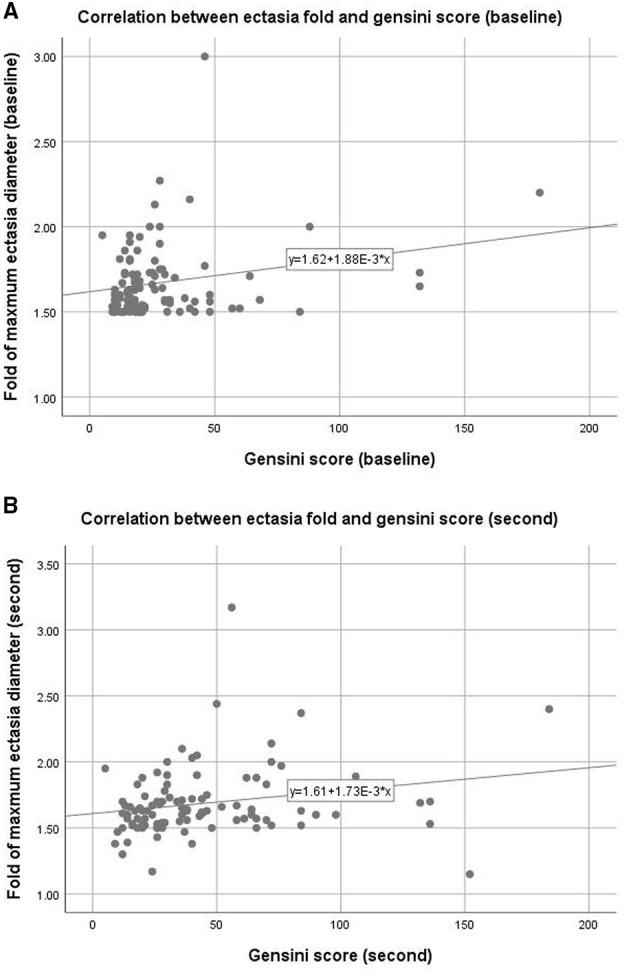
Correlation between coronary ectasia and stenosis. Upper panel **(A)**: Baseline data measured from the first coronary angiogram, Pearson correlation coefficient = 0.227, *p* = 0.024, R^2^ = 0.048. Lower panel **(B)**: follow-up data measured from the second coronary angiogram, Pearson correlation coefficient = 0.214, *p* = 0.033, R^2^ = 0.072.

Figures 2A,B show the Pearson correlation analysis of the relationship between ectasia diameter and Gensini scores for the first and t second CAGs. Gensini scores had a significant relationship with ectasia diameter at both time points (Pearson correlation coefficients and *p*-values were: 0.227, 0.024, 0.214, and 0.033, respectively).

**Figure 3:**
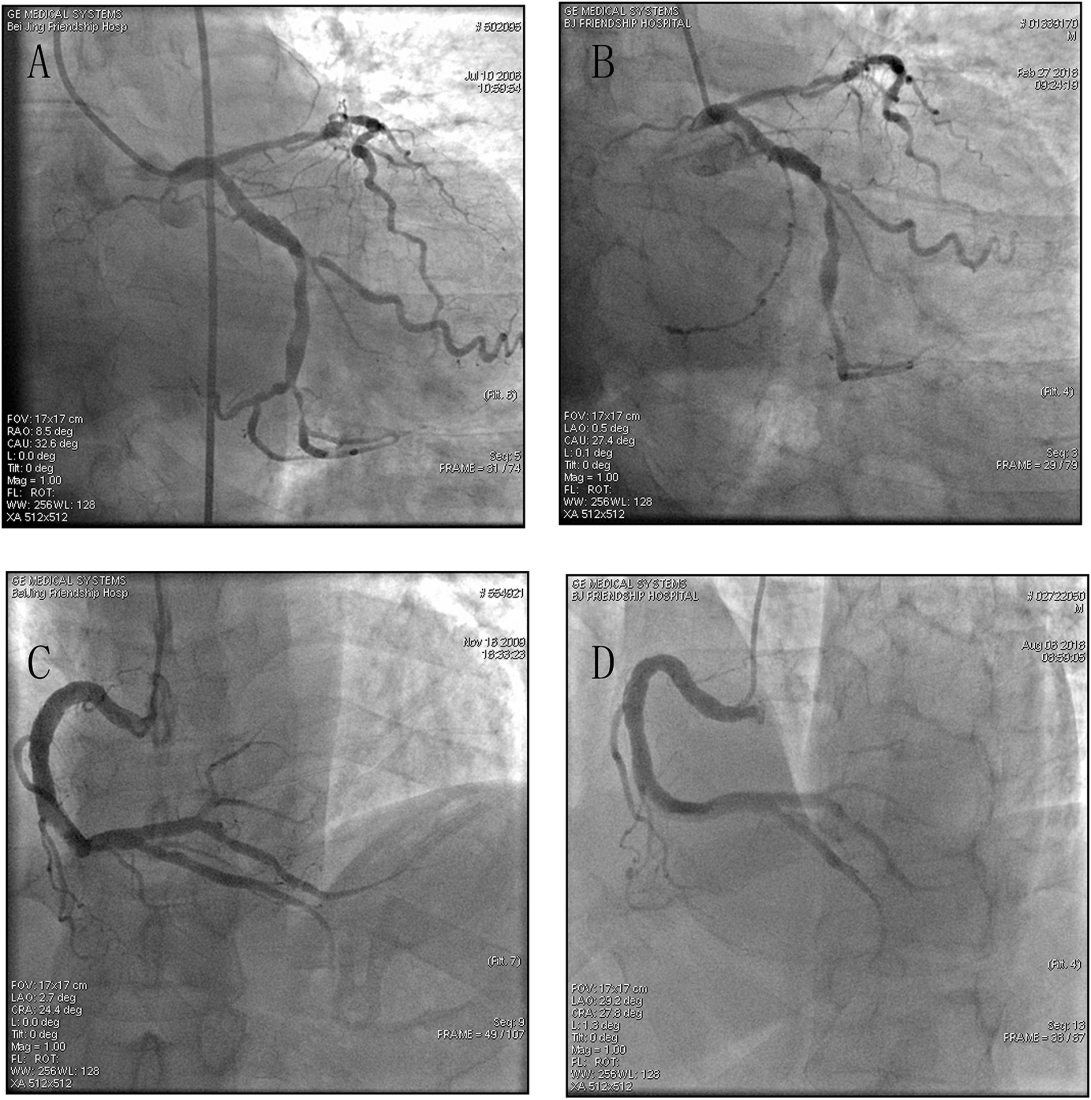
Typical CAG images from two CAE patients. The tube in each figure at the opening of vessel was a catheter used for operating CAG. Its diameter was 5 French (1 French = 1/3 mm) and it could be used as a marker for measuring the diameter and length of ectatic changes. **(A)** (first CAG) and **(B)** (second CAG): 9 years follow-up for a female CAE patient with diffused dilation in right coronary artery (RCA), the maximum diameter of RCA was 4.94 folds of the catheter or 8.23 mm or at least 1.64 folds of normal RCA (normal RCA was <5 mm); **(C)** (first CAG) and **(D)** (second CAG):12 years follow-up for a male CAE patient with segmental dilation in left circumflex coronary artery (LCX), the maximum diameter of LCX was 3.46 folds of the catheter or 5.76 mm or 1.76 folds of normal vessel.

In Figure 3, typical CAG images from two CAE patients showed there were minimal changes along with time for ectasia extent.

## Discussion

This self-controlled retrospective cohort study mainly explored whether CAE was a progressive disease by comparing clinical features and imaging data between patients' first and second CAG examinations. The results revealed that: (1) All CAE patients had evidence of atherosclerosis, and a majority presented with AMI. Most of the basic characteristics increased over time except blood pressure and lipid concentrations, which had decreasing tendencies. (2) There were no significant changes in inflammation indicators, but there were small changes in heart function including increases in left atrium dimensions and fractional shortening percentages. (3) Atherosclerosis increased significantly as indicated by the distribution of stenosis in coronary arteries and Gensini scores. However, the ectasia extent showed minimal change as reflected by blood vessel involvement, Markis type, ectatic diameter, ectatic length, and coronary blood flow. (4) Multilinear regression analysis revealed that the underlying factors for stenosis changes (as indicated by the fold change of Gensini score) were longer follow-up time, lower baseline Gensini scores, and higher hypersensitive C-reactive protein concentrations (5). The RCA was most frequently involved in the ectatic changes. The percentage of patients with both stenosis and ectasia in the RCA was higher after the second CAG. The ectatic diameter was related to the extent of stenosis, as indicated by the Gensini score at both CAG time points. The self-comparisons for subgroups including CAE with AMI and without AMI, as well as CAE with revascularization and without revascularization, were also applied and there were no changes for ectatic extent while the extent of stenosis progressed (Supplementary Material).

The first major finding was that there were no significant changes in ectatic extent and coronary blood flow in either follow-up group of 1–5 years (time interval, 3.04 [2.48–4.17] years) and 5–16 years (time interval, 8.15 [6.52–11.48] years). These results are clinically significant for several reasons. (1) CAE patients might have a longer natural history than presumed if their condition progressed very slowly. They might have experienced coronary ectatic changes at younger ages, although the average age at diagnosis was 55 ± 10 years (2). (2) CAE seemed to have a more intimate relationship with genetic abnormalities in patients who presented with coronary dilation at a younger age and did not have Kawasaki disease or other pathogenic factors (e.g., infections, percutaneous coronary intervention, etc.) (18). (3) Because of the slow progression of ectasia, one of major dangerous outcome might not be continuous rapid expansion even the coronary rupture at the ectatic sites like giant coronary artery aneurysm, which is currently considered an important issue by some researchers (15, 19). CAE might be different from aneurysms in the brain and aortic artery, which are prone to rupture and thus need to be remedied as soon as possible. Rather, the greatest damage might be thrombosis and slow blood flow in the coronary artery, which would result in AMI and unstable angina pectoris (20). (4) Percutaneous coronary intervention, especially stent implantation in the ectatic site, is seldom considered because of concerns about stent migration due to continuous dilation in patients with coronary ectasia (21). Our results suggest that stent implantation might be acceptable in CAE patients if the coronary was coexisted with ectasia and CAD.

The second important finding was significant progression of coronary atherosclerotic changes in CAE patients. Part of the patients were received stent implant outside of ectatic site after first CAG (Supplementary Material), their atherosclerotic changes also progressed while ectatic changes did not (Supplementary Material)… An important question is why CAE was not diagnosed at a younger age if it is a chronic progressive disease. A possible reason is that the clinical manifestations of CAE (e.g., angina, AMI, arrhythmia, and sudden death) might originate from not just ectasia but also other pathological changes such as stenosis progression. It is likely that there were no obvious clinical manifestations at the early stage because the stenosis was slight or absent. However, with atherosclerotic progression, the patients eventually developed the above-mentioned symptoms and were then diagnosed by CAG or other imaging techniques. Thus, the prevention and treatment of atherosclerotic changes might have more clinical significance than the prevention and treatment of ectatic changes. Indeed, there are no universally accepted treatment strategies for CAE. Most proposals are based on the treatments for CAD (22, 23). Antithrombotic agents, statins and anti-ischemic therapies are the main treatments because most CAE cases are associated with CAD, and approximately 50% of patients experience angina symptoms (24–26). In the present study, all CAE patients exhibited atherosclerosis, 48.48% (48/99) had experienced AMI, and 45.45% (45/99) had unstable angina pectoris. The AMI rate in CAE population was higher than in CAD patients (20), in those patients atherosclerotic changes also progressed while ectatic changes did not (Supplementary Material), there is no consensus which was the best anti-thrombotic strategy as the single antiplatelet agent, dual antiplatelet therapy (DAPT) or anti-coagulant therapy currently, CAE patient with larger coronary diameter and recurrent thrombotic event might require anti-coagulant therapy (27). The study results thus provide additional evidence to support the current CAD-based treatment strategy for CAE patients.

The relationship between CAE and CAD remains unclear. We observed that most ectatic changes were anatomically accompanied by stenosis, especially in the RCA. There was a significant relationship between ectasia and stenosis. There are two possible explanations for the relationship between CAE and CAD. The first is that CAE is a variation of CAD (14), with more than 80% of CAE patients also presenting with CAD (3, 28). CAE patients experience atherosclerotic changes in the intimal–media interface of the coronary artery. The vessel wall is infiltrated by several types of inflammatory cells (neutrophils, lymphocytes, plasma cells, eosinophils, and mononuclear cells) (8). It has therefore been suggested that the two diseases might share a pathological process and that CAE is a variation of CAD. The other standpoint, CAE is a risk factor for CAD for several reasons. (1) Pathologically, CAE is characterized by significant damage in the smooth muscle and elastic fiber layers of the coronary artery, while CAD is not (4). (2) Clinical epidemiology data demonstrated that most CAE patients experience CAD while few CAD patients exhibit CAE. In a large population-based survey with 10,524 cases, 87% of the CAE patients had CAD, but only 3% of CAD patients exhibited CAE (20). (3) The two diseases also have different progression rates. We found that ectatic changes occurred over a longer period in patient with CAE compared to those with CAD. Therefore, we prefer that CAE might present before CAD and is more likely to be an underlying risk factor for CAD progression. This hypothesis requires further exploration.

### Limitations

Given the rarity of CAE, it was not easy to observe disease progression in a large patient cohort. Our work has other limitations. Since it was a single-center study, further multi-center collaborations are needed to produce more representative results. We were unable to assess natural CAE progression and dynamic changes because most of the enrolled subject received medications and underwent revascularization procedures. Ethics rules preclude the design clinical trials without any treatments. Thirdly, we did not fully assess the occurrence of major adverse cardiovascular events during follow-up period, such as stroke, recurrent myocardial infarction, heart failure, cardiovascular death, and left ventricular dysfunction. Fourthly, this study mainly enrolled the patients who undertook at least two times of CAG evaluations retrospectively, the dynamic changes in coronary for the patient who did not undertake or had no chance to receive a second CAG were not observed. More work is needed to understand pathological changes in CAE.

### Conclusion

Our study shows that ectatic changes in CAE patients occurred relatively slowly over a period of 1–16 years. However, there was evidence of atherosclerotic progression. The results indicated the prevention and treatment of atherosclerotic changes might have more clinical significance than addressing ectatic changes. Additional investigations are needed to confirm these conclusions and deductions, particularly because of the possibility of bias resulting from the small sample size and sample enrollment of this retrospective cohort study.

## Data Availability Statement

The data analyzed in this study is subject to the following licenses/restrictions: extra data is available by emailing Siwen Liang. Requests to access these datasets should be directed to Siwen Liang, 278713478@qq.com.

## Ethics Statement

This study was approved by the Ethics Committee of Beijing Friendship Hospital Affiliated to Capital Medical University and was in accord with the Declaration of Helsinki (P2-013-02). Informed consent was not obtained from all the study participants because it was a retrospective research and no extra medical interventions were introduced for all subjects.

## Author Contributions

RL and SL: conceived the study and its design, conceptualization, formal analysis, resources, and software. SL: data curation. RL, XG, HZ, and SL: investigation and validation. XG: methodology. RL, HZ, and SL: project administration. All authors contributed to the article and approved the submitted version.

## Funding

This study was sponsored by the National Natural Science Foundation of China (Grant No. 81600276).

## Conflict of Interest

The authors declare that the research was conducted in the absence of any commercial or financial relationships that could be construed as a potential conflict of interest.

## Publisher's Note

All claims expressed in this article are solely those of the authors and do not necessarily represent those of their affiliated organizations, or those of the publisher, the editors and the reviewers. Any product that may be evaluated in this article, or claim that may be made by its manufacturer, is not guaranteed or endorsed by the publisher.

## Abbreviations

BMI: body mass index
CAD: coronary artery disease
CAE: coronary artery ectasia
CAG: coronary angiography
LAD: left anterior descending coronary artery
LCX: left circumflex coronary artery
LM: left main coronary artery
RCA: right coronary artery.
