# Supplementary material for: Is Coronary Artery Ectasia a Progressive Disease? A Self-Controlled Retrospective Cohort Study
Source: Front Cardiovasc Med. 2021 Dec 6;8:774597. doi: 10.3389/fcvm.2021.774597 (PMC8685394; doi:10.3389/fcvm.2021.774597)
Supplement: Supplementary file 1 [file Data_Sheet_1.pdf]

Liner regression for ectasia

Most of the dilated coronaries had no significant changes along times according to our practices. The folds of ectaisa extent including diameters, length, and CTFC), the detail data were listed in following tables. As those comparisons between the two CAGs were in no significance and those values for the fold between the two CAGs were not very big, thus after a discussion the further regression was not presented in the manuscript.

		Mean value	median value	Standard deviation	minimum value	Maximum value	Percentile		
							25%	50%	75%
**Interval 1–5 years (n = 53)**	Fold of ectasia diameter	0.993	1.000	0.072	0.760	1.110	0.977	1.000	1.049
	Fold of ectasia length	1.002	1.000	0.120	0.530	1.400	0.980	1.000	1.036
	Fold of CTFC	1.024	1.000	0.183	0.580	1.500	0.889	1.000	1.133
	Fold of gensini	1.548	1.286	0.747	0.880	4.670	1.100	1.286	1.701
**Interval 5–16 years (n = 46)**	Ectasia diameter	1.027	1.021	0.107	0.700	1.360	0.977	1.021	1.068
	Ectasia length	1.005	1.012	0.127	0.660	1.490	0.926	1.012	1.075
	Fold of CTFC	1.111	1.043	0.306	0.570	2.000	0.900	1.043	1.227
	Fold of gensini	1.884	1.392	0.918	1.000	4.400	1.200	1.392	2.433

Here the multiple linear regression for the changes of ectasia listed in following table was not hard to analyze, we did not embed them into the manuscript.

Items	Beta	Standardization beta coefficient	95% CI for beta		*t*-value	*p*-value
Constant	1.190		1.073	1.306	20.628	0.000
Age, years	-0.002	-0.372	-0.004	-0.001	-2.718	0.010
Statins, ***n*** **(%)**	-0.035	-0.240	-0.075	0.005	-1.748	0.088
Gensini score	-0.001	-0.489	-0.002	-0.001	-3.833	0.000

The dependent factor was ectaisa changes between the first and second coronary angiograms (CAGs). The independent factors, which were selected from the first CAG, were sex, age, hypertension, diabetes, smoking, alcohol, low density lipoprotein cholesterol, hypersensitive C-reactive protein, Markis classification, ectasia extent, baseline Gensini score, follow-up time, antiplatelet, statins, angiotensin-converting enzyme inhibitors and angiotensin receptor blockers, beta blockers, calcium channel blockers and nitrates.

We have try to make regressions for both the ectasia changes and the stenosis changes. Eventually just the regression for stenosis changes was implemented and presented in the manuscript (in table 5) because a significant changes between the two CAGs were observed.
