# Supplementary material for: Is Coronary Artery Ectasia a Progressive Disease? A Self-Controlled Retrospective Cohort Study
Source: Front Cardiovasc Med. 2021 Dec 6;8:774597. doi: 10.3389/fcvm.2021.774597 (PMC8685394; doi:10.3389/fcvm.2021.774597)
Supplement: Supplementary file 2 [file Data_Sheet_2.pdf]

Information for stent implant

There were 62 CAE patients received stent implant, and 54 of them received 1 stent, 5 of them received 2 stents, 2 of them received 3 stents, 1 of them received 4 stents. Thus total 84 stents were implanted. The size of stent was different in different vessel, thus the average diameter of stents would be presented separately according to their location in coronary.

The main branches of coronary include: left main coronary artery (LM), left anterior descending branch (LAD), diagonal branches(D), left circumflex branch(LCX), obtuse marginal branch(OM), right coronary artery(RCA), posterior descending branches artery(PDA), posterior branches artery of left ventricular(PLA).

The coronary could be divided into proximal segment (p), middle segment (m), distal segment (d), and terminal branches. For example, LADp means proximal segment of LAD. LAD and RCA have all of the three segments; LM and LCX only have proximal and distal segments; D, OM, PLA, PDA were not further divided usually. In this research, no stents were implanted in LM and D.

The detailed data were as following:

Vessel	Segment	Stent name	Stent diameter (mm)	Stent length (mm)	Average diameter(mm)	Average length (mm)
LAD	LADp	S7	3.50	18.00	3.27	22.04
	LADp	Firebird	3.50	18.00		
	LADp	Excel	3.50	18.00		
	LADp	Excel	3.00	14.00		
	LADp	Excel	3.50	18.00		
	LADp	Endeavor	3.00	18.00		
	LADp	Cypher	2.50	18.00		
	LADp	Taxus	4.00	23.00		
	LADp	Cypher	3.00	23.00		
	LADp	Taxus	3.50	24.00		
	LADp	Excel	3.50	28.00		
	LADp	Resolute	4.00	30.00		
	LADp	Endeavor	2.50	30.00		
	LADp	Excel	3.50	24.00		
	LADp	Xience	3.00	23.00		
	LADp	Partner	2.75	21.00		
	LADp	Excel	3.50	36.00		
	LADp	Endeavor	3.00	3.00		
	LADp-m	Xience	3.00	28.00		
	LADp-m	Resolute	4.00	30.00		
	LADp	Taxus	3.00	32.00		

	LADp	Cypher	3.00	18.00		
	LADo-p	Taxus	3.50	12.00		
	LADm	S7	3.00	18.00	2.99	26.47
	LADm	Firebird	3.00	33.00		
	LADm	Taxus	2.50	12.00		
	LADm	Cypher	3.50	18.00		
	LADm	Taxus	3.00	33.00		
	LADm	Partner	2.75	24.00		
	LADm	Xience	3.00	38.00		
	LADm	Partner	3.50	24.00		
	LADm	Xience	3.50	28.00		
	LADm	Partner	2.75	36.00		
	LADm	Endeavor	3.00	30.00		
	LADm	Endeavor	3.00	15.00		
	LADm	Excel	3.00	36.00		
	LADm	Partner	2.50	18.00		
	LADm	Partner	2.75	36.00		
	LADm	Taxus	3.00	33.00		
	LADd	Excel	3.00	18.00	3.00	18.00
LCX	LCXp	S7	3.50	18.00	3.14	25.71
	LCXp	Taxus	4.00	32.00		
	LCXp	Endeavor	2.50	18.00		
	LCXd	Partner	4.00	36.00		
	LCXp	Endeavor	2.50	30.00		
	LCXp	Endeavor	3.00	30.00		
	LCXp	Partner	2.50	16.00		
	LCXd	Firebird	3.00	29.00	2.68	22.71
	LCXd	Endeavor	2.50	18.00		
	LCXd	Endeavor	2.50	18.00		
	LCXd	Partner	2.75	29.00		
	LCXd	Partner	2.50	29.00		
	LCXd	Resolute	2.75	18.00		
	LCXd	Endeavor	2.75	18.00		
	LCXd	Firebird	3.00	29.00		
	LCXd	Endeavor	2.50	18.00		
	LCXd	Endeavor	2.50	18.00		
	LCXd	Partner	2.75	29.00		
	LCXd	Partner	2.50	29.00		
	LCXd	Resolute	2.75	18.00		
	LCXd	Endeavor	2.75	18.00		
	OM	S8	2.50	12.00	2.60	16.80
	OM	Vision	2.75	18.00		
	OM	Endeavor	2.50	18.00		

	OM	Endeavor	2.50	18.00		
	OM	Endeavor	2.75	18.00		
RCA	RCAp	Taxus	4.00	32.00	4.00	28.00
	RCAp	Endeavor	4.00	18.00		
	RCAp-m	Xience	4.00	38.00		
	RCAp	Resolute	4.00	24.00		
	RCAm	Cypher	3.50	23.00	3.10	26.20
	RCAm	Excel	3.50	18.00		
	RCAm	Excel	3.50	18.00		
	RCAm	Excel	2.50	36.00		
	RCAm	Excel	2.50	36.00		
	RCAd	Cypher	3.50	23.00	2.69	25.50
	RCAd	Excel	2.50	36.00		
	RCAd	Xience	2.50	15.00		
	RCAd	Xience	2.25	28.00		
	PLA	Excel	2.50	18.00	2.67	18.00
	PLA	Endeavor	2.75	18.00		
	PLA	Endeavor	2.75	18.00		
	PDA	Partner	2.75	21.00	3.13	19.50
	PDA	Endeavor	3.50	18.00		
