# Supplementary material for: Is Coronary Artery Ectasia a Progressive Disease? A Self-Controlled Retrospective Cohort Study
Source: Front Cardiovasc Med. 2021 Dec 6;8:774597. doi: 10.3389/fcvm.2021.774597 (PMC8685394; doi:10.3389/fcvm.2021.774597)
Supplement: Supplementary file 3 [file Data_Sheet_3.pdf]

Comparisons for subgroups

Part of the patients were received treatment of revascularization (or treatment of stent implant, we did not give other PCI treatments) and we have not located the stent in the ectasia part because it was absent of evidences. Also the patients who were received CABG were excluded.

We divided the patients into two groups according to the treatment with revascularization or without revascularization, the result was as same as the table 3 showed in the manuscript. The key results were listed as following:

	Without revascularization n=37			With revascularization n=62		
	Baseline	Second	p-value	Baseline	Second	p-value
Ectasia diameter, mm	5.12± 0.97	5.05± 1.11	0.386	5.20± 0.95	5.31± 1.03	0.521
Ectasia length, mm	12.04± 6.20	11.82± 6.22	0.341	13.17± 7.07	13.37± 7.43	0.879
Ectasia fold	1.64± 0.17	1.62± 0.24	0.437	1.69± 0.24	1.73± 0.28	0.436
CTFC	31.30(25.00-35.83)	30.00(26.00-35.30)	0.346	32.00(26.95-40.00)	32.45(30.00-40.00)	0.551
Gensini score	18.00(12.00-30.00)	28.00(16.00-52.00)	0.000	21.00(16.00-30.25)	38.00(25.50-63.00)	0.000

By adding the revascularization into the regression model for change of Gensini score, it could be found that the revascularization was excluded by the model. The regression model after adding revascularization into dependent factors was as the same as before adding revascularization into dependent factors.

**Items**	**Beta**	**Standardization beta coefficient**	**95% CI for beta**		***t*-value**	***p*-value**
Constant	1.257		0.850	1.664	6.171	0.000
Follow-up time (month)	0.005	0.228	0.000	0.010	2.023	0.047
Gensini score	−0.006	−0.217	−0.011	0.000	−1.936	0.057
Hs-CRP, mg/L	0.046	0.286	0.010	0.083	2.539	0.014

Also in this research some CAE patients were with AMI, some were without AMI, we also divided the patients into two groups according to be with AMI or without AMI,

the result was as same as the table 3 showed in the manuscript. The results were as following:

	Without AMI n=51			With AMI n=48		
	Baseline	Second	p-value	Baseline	Second	p-value
Ectasia diameter, mm	5.12± 1.06	5.11± 1.18	0.860	5.23± 0.83	5.33± 0.92	0.055
Ectasia length, mm	11.36± 5.43	11.35± 5.48	0.926	14.22± 7.70	14.33± 8.12	0.554
Ectasia fold	1.68± 0.26	1.67± 0.32	0.857	1.67± 0.17	1.70± 0.21	0.069
CTFC	32.00(28.00-40.00)	32.00(28.00-36.00)	0.787	32.00(24.00-40.00)	32.00(28.00-40.00)	0.302
Gensini score	18.00(13.00-29.00)	30.00(17.00-46.00)	0.000	21.50(16.50-31.50)	39.00(26.00-70.00)	0.000
